# Chemotaxis and Related Signaling Systems in *Vibrio cholerae*

**DOI:** 10.3390/biom15030434

**Published:** 2025-03-18

**Authors:** Fuga Omori, Hirotaka Tajima, Sotaro Asaoka, So-ichiro Nishiyama, Yoshiyuki Sowa, Ikuro Kawagishi

**Affiliations:** 1 Department of Frontier Bioscience, Hosei University, 3-7-2 Kajino-cho, Koganei City, Tokyo 184-8584, Japan; fuga.omori.8w@stu.hosei.ac.jp (F.O.); hirotaka.tajima.2n@hosei.ac.jp (H.T.); sotaro.asaoka.7k@stu.hosei.ac.jp (S.A.); ysowa@hosei.ac.jp (Y.S.); 2 Research Center for Micro-Nano Technology, Hosei University, 3-11-15 Midori-cho, Koganei City, Tokyo 184-0003, Japan; 3 Faculty of Applied Life Science, Niigata University of Pharmacy and Medical and Life Sciences, 265-1 Higashijima, Akiha-ku, Niigata City, Niigata 956-8603, Japan; snishiyama@nupals.ac.jp

**Keywords:** signal transduction, bacterial flagella, flagellar motor, motility, chemotaxis, receptor, two-component system, pathogenicity

## Abstract

The motility and chemotaxis of *Vibrio cholerae*, the bacterial pathogen responsible for cholera, play crucial roles in both environmental survival and infection. Understanding their molecular mechanisms is therefore essential not only for fundamental biology but also for infection control and therapeutic development. The bacterium’s sheathed, polar flagellum—its motility organelle—is powered by a sodium-driven motor. This motor’s rotation is regulated by the chemotaxis (Che) signaling system, with a histidine kinase, CheA, and a response regulator, CheY, serving as the central processing unit. However, *V. cholerae* possesses two additional, parallel Che signaling systems whose physiological functions remain unclear. Furthermore, the bacterium harbors over 40 receptors/transducers that interact with CheA homologs, forming a complex regulatory network likely adapted to diverse environmental cues. Despite significant progress, many aspects of these systems remain to be elucidated. Here, we summarize the current understanding to facilitate future research.

## 1. Introduction

**Figure 1:**
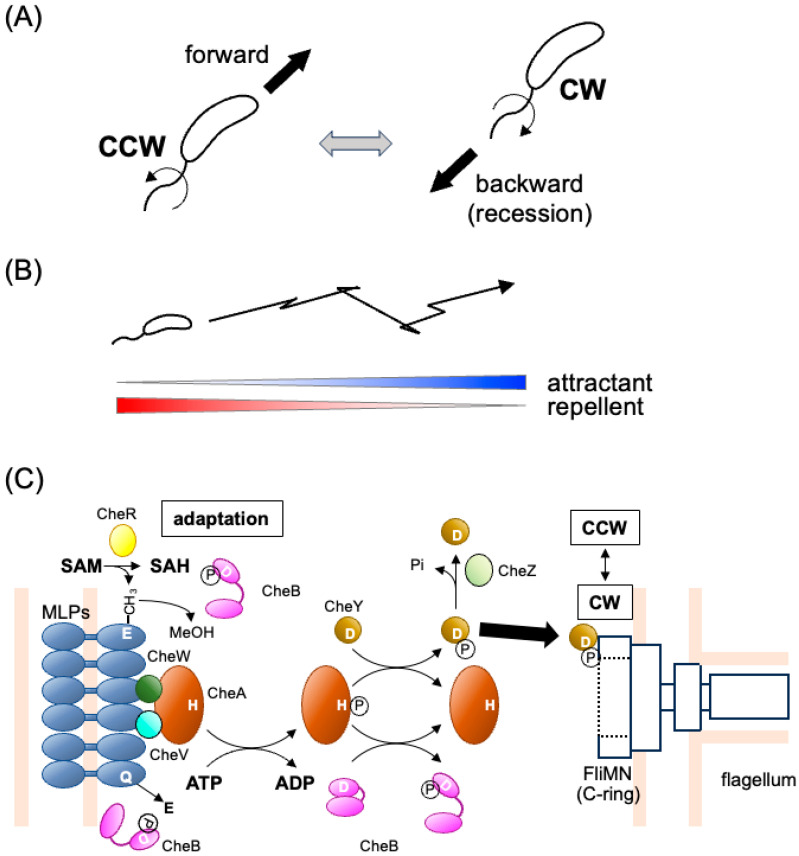
Single polar flagellum and swimming behavior of *V. cholerae*. (**A**) *V. cholerae* navigates liquid environments by rotating its single polar flagellum. The direction of rotation dictates its swimming behavior: counterclockwise (CCW) rotation propels the bacterium forward, while clockwise (CW) rotation pulls it backward. (**B**) The bacterium exhibits chemotaxis by modulating the rotational direction of its flagellar motor in response to environmental cues. This motor switching enables movement along gradients of attractants and repellents. (**C**) The chemotaxis signaling system of *V. cholerae*. Various environmental stimuli, such as amino acids and taurine, are detected by MLPs (MCP-like proteins) (blue). These MLPs form complexes with the histidine kinase CheA (brown) and adaptor proteins CheW (dark green) and CheV (light blue). Binding of an attractant deactivates CheA, whereas binding of a repellent activates it. The auto-phosphorylated CheA then transfers its phosphoryl group to the response regulator CheY (ochre). Phosphorylated CheY binds to the switch proteins FliM and FliN, which constitute the cytoplasmic ring of the flagellar motor, thereby inducing a switch to the CW rotation (the default rotation being CCW). CheZ (light green) facilitates the dephosphorylation of CheY. During adaptation, specific glutamate residues in typical MLPs are methylated by the methyltransferase CheR (yellow) and demethylated by the methylesterase CheB (magenta), which also exhibits deamidase activity on specific glutamine residues and is activated by phosphorylation from CheA.

*Vibrio cholerae*, the causative agent of cholera, is a highly motile, Gram-negative bacterium with a single flagellum extending from one pole of its curved, rod-shaped cell (Figure 1A). This bacterium thrives in both nutrient-poor aquatic environments, such as rivers and estuaries, and nutrient-rich gastrointestinal environments within host animals [1,2].

Chemotaxis—the ability of *V. cholerae* to migrate toward favorable conditions (Figure 1B)—is thought to play a crucial role in its survival across diverse environments [3]. Additionally, both motility and chemotaxis have been implicated in the bacterium’s pathogenicity [4,5,6,7,8,9,10]. However, our current understanding of the molecular mechanisms underlying these processes and their physiological significance remains limited. In this brief review article, we summarize the existing knowledge on *V. cholerae* motility and chemotaxis, incorporating some of our unpublished findings.

## 2. A Brief Overview of Motility and Chemotaxis of *Vibrio cholerae*

### 2.1. Single, Sheathed Polar Flagellum

Many bacteria achieve motility by rotating their flagella, enabling movement toward favorable environments [11,12,13]. Each flagellum extends from the cell body and consists of a long, tubular, helical filament that functions like a propeller, driven by a reversible rotary motor at its base. Depending on the species, this motor is powered by the flux of either protons or sodium ions across the cytoplasmic membrane. The motor can switch between counterclockwise (CCW) and clockwise (CW) rotation (viewed from the filament toward the motor), allowing the bacterium to navigate its surroundings. This switching behavior is regulated by the chemotactic protein CheY in an intracellular two-component regulatory system (see Section 2.2). The best-studied examples of flagellar motility come from peritrichously flagellated enteric bacteria, such as *Escherichia coli* and *Salmonella enterica* serovar Typhimurium. In these bacteria, CCW motor rotation aligns the flagellar filaments into a bundle, enabling smooth swimming, whereas CW rotation disrupts the bundle, causing the cell to tumble and change direction. In chemotaxis, bacteria navigate chemical gradients by modulating the balance between smooth swimming and tumbling, allowing them to move toward attractants and away from repellents.

*V. cholerae* possesses a single, sheathed flagellum located at one cell pole (for review, see [14]). The number of flagella is strictly regulated by the opposing actions of the positive regulator FlhF [15,16] and the negative regulator FlhG [15]. While the flagellar sheath appears to be continuous with the outer membrane, its protein composition is distinct from lipopolysaccharides, suggesting that it is not merely an extension of the outer membrane [17]. Other *Vibrio* species, such as *V. parahaemolyticus* and *V. alginolyticus*, also possess a single, sheathed polar flagellum, in addition to the lateral peritrichous flagella [14]. The *Vibrio* polar flagellum shares conserved genes, structural features, and functions across species and is powered by the sodium ion motive force across the membrane [18,19]. It rotates in both clockwise (CW) and counterclockwise (CCW) directions, propelling the cell backward and forward, respectively (Figure 1A). Interestingly, the flagellum can bend, generating a flicking motion that alters the cell’s movement pattern and facilitates chemotaxis [20,21].

The flagellum of *V. cholerae* is essential not only for motility but also for attachment and colonization, both of which are critical for infection [22,23]. Unlike unsheathed flagella, such as those of *S. enterica* serovar Typhimurium, the *V. cholerae* flagellum is less prone to the disassembly and shedding of flagellin monomers—the primary structural components of flagella. This reduces the activation of the innate immune response, specifically NF-κB activation in human epithelial cells [24]. Beyond its roles in motility and immune evasion, the *V. cholerae* flagellum also functions as a secretion channel for virulence factors. For example, the bacterial cytotoxin MakA is secreted through the flagellar channel in a proton-motive-force-dependent manner [25]. The multifunctionality of the flagellum underscores its importance in both environmental survival and pathogenicity.

### 2.2. Chemotaxis and Related Signaling Systems

Chemotaxis signaling systems are specialized variations of the two-component regulatory system (TCS; for reviews, see [26,27]), with the histidine kinase CheA and the response regulator CheY serving as the central processing unit (Figure 1C; for review, see [28] and references therein). Unlike most TCSs, chemotaxis systems are unique in that CheA is not a direct sensor but instead interacts with distinct receptor/transducer proteins. These receptors, known as methyl-accepting chemotaxis proteins (MCPs) or chemoreceptors, belong to a family of transmembrane and occasionally cytoplasmic proteins. MCPs form a ternary complex with the adaptor protein CheW and the histidine kinase CheA, assembling into an organized array at a cell pole [29,30,31,32] (see Section 4.2). CheA activation occurs when it is associated with chemoreceptors that are not bound by attractants. Upon autophosphorylation, CheA transfers its phosphoryl group to CheY, which then binds to the switching components of the flagellar motor, inducing clockwise (CW) rotation. When an attractant binds to MCPs, CheA activity is inhibited, leading to a decrease in the cellular concentration of phosphorylated CheY (phospho-CheY) and promoting counterclockwise (CCW) flagellar rotation. Adaptation to changing environmental signals involves two additional regulatory proteins—the methylesterase CheB and the methyltransferase CheR. CheR catalyzes the methylation of specific glutamate residues on MCPs (Glu to mGlu) using S-adenosyl methionine, while CheB hydrolyzes methylated glutamate residues, restoring them to their original form (mGlu to Glu). Additionally, CheB functions as a deamidase, converting glutamine residues to glutamate (Gln to Glu) and thus further fine-tuning receptor sensitivity. While *V. cholerae* follows the general chemotaxis signaling paradigm, its system is notably more complex.

**Figure 2:**
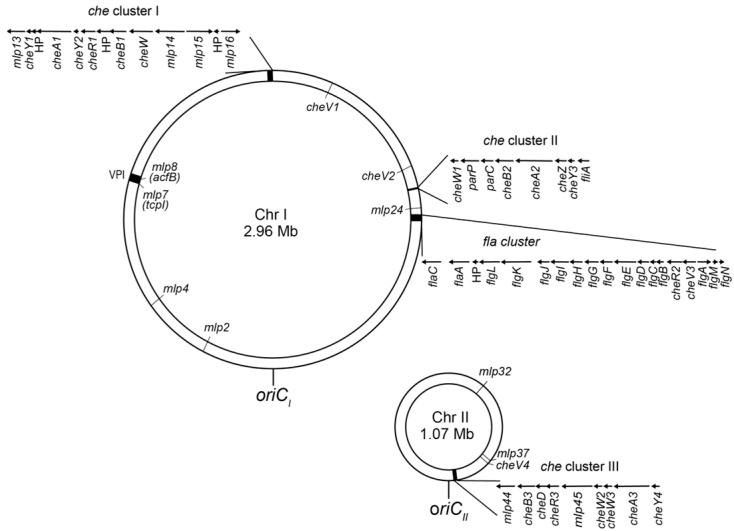
Genes for flagellation, motility, and chemotaxis in *V. cholerae*. *V. cholerae* has two chromosomes: Chromosome I (Chr I) and Chromosome II (Chr II). The *fla* gene cluster and the three *che* gene clusters, as well as the *Vibrio* pathogenicity island (VPI), are shown in black. Sizes and polarities of the clustered genes are shown as arrows. Positions of the replication origins (*oriC_I_* and *oriC_II_*) and the non-clustered *che* genes, as well as some of the non-clustered *mlp* genes of interest, are indicated.

The genome sequencing of *Vibrio cholerae* (Classical and El Tor biotypes) reveals that the bacterium possesses a single set of flagellar biosynthesis and motility genes but three distinct sets of chemotaxis signaling proteins [33,34] (Figure 2). The genes encoding each chemotaxis protein set are organized into clusters within the genome, where Clusters I and II are located on the larger chromosome, while Cluster III resides on the smaller chromosome.

Cluster I: *cheY1*, *cheA1*, *cheY2*, *cheR1*, *cheB1*, and putative *cheW*
Cluster II: *cheW1*, *cheB2*, *cheA2*, *cheZ* and *cheY3*
Cluster III: *cheB3*, *cheD*, *cheR3*, *cheW2*, *cheW3*, *cheA3* and *cheY4*.

The *cheZ* gene product facilitates the dephosphorylation of phospho-CheY [35,36], while the *cheD* gene product catalyzes the deamidation of specific glutamine residues on MCPs (Gln to Glu) [37]. Additionally, the *cheR2* gene is located within the *fla* gene cluster, adjacent to the *che* Cluster II. In some bacteria, the receptor array requires not only CheA and CheW but also CheV, a CheW-like adaptor protein [38,39]. *V. cholerae* possesses four genes encoding CheV homologs [39,40]. Among them, CheV1 is implicated in chemotaxis [40]. CheV4 also integrates into the receptor cluster in the absence of CheA, which suggests that CheV4 modulates receptor function under certain conditions [39].

**Figure 3:**
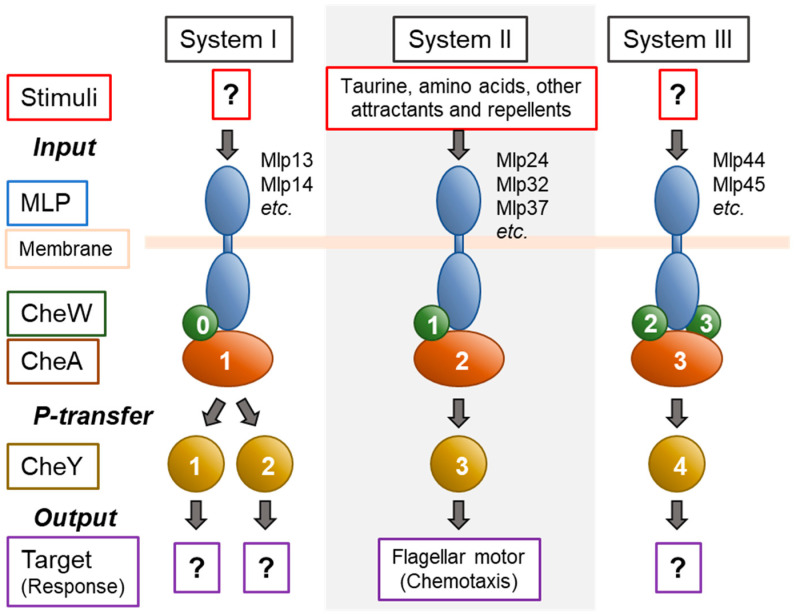
Chemotaxis and related signaling systems of *V. cholerae*. The MLPs and the Che proteins of *V. cholerae* are considered to be organized into three parallel signaling systems, of which System II is the only one proven to be responsible for chemotaxis. Note that CheW of System I is marked with "0".

The fact that most of the genes encoding homologs of various Che proteins are located in three separate clusters on the genome predicts that these proteins (and other proteins encoded by the genes outside of the clusters) constitute three distinct signaling systems named Che Systems I, II, and III (Figure 3). Out of these systems, only System II has been proven to be involved in chemotaxis [33,41]. The other two systems are probably involved in other cellular functions or in chemotaxis in specific environments. However, their roles have been elusive since neither the deletion nor the overexpression of genes of CheY homologs of these systems give rise to any detectable phenotype [41]. It should be noted that CheA1, CheW, and CheY2 of System I are localized to the cell poles under oxygen-limiting conditions [42], suggesting that System I components are assembled into the supramolecular signaling complex in response to reduced cellular energy states. Furthermore, Mlp45 (Aer2), belonging to System III, is presumed to be an O_2_ sensor [43]. It is tempting to assume that Systems I and III are involved in sensing and signaling under microaerobic environments, such as in the host intestine.

### 2.3. MLPs (Methyl-Accepting Chemotaxis Protein-like Proteins)

Genome sequencing of *Vibrio cholerae* predicts the presence of 46 MCP-like proteins (hereafter referred to as MLPs) [34]. In the El Tor strain, 45 *mlp* genes are designated *mlp1* through *mlp45* based on their chromosomal locations (Table S1) [44]. The classical strain genome contains 44 *mlp* genes, lacking *mlp5* and *mlp6* but possessing an additional gene, *mlp46* (NCBI accession: GCF_000016245.1, GCA_000021625.1).

MLPs are characterized by a conserved 48-residue sequence, known as the highly conserved domain (HCD) [45,46] (see Section 4.1). In the cytoplasmic regions, the HCD is flanked by coiled-coil regions resembling the methylation helices of canonical MCPs, although their lengths vary from 24 to 46 residues. Based on membrane topology, MLPs can be classified into the following three types: (i) MLPs with two predicted transmembrane (TM) regions per subunit, featuring a putative periplasmic domain between them, (ii) MLPs with two TM regions but lacking a periplasmic domain, and (iii) cytoplasmic MLPs with no predicted TM regions.

Within the chemotaxis gene clusters, *mlp13*, *mlp14*, *mlp15*, and *mlp16* are found in Cluster I, while *mlp44* and *mlp45* are in Cluster III, suggesting that these genes encode system-I and system-III MLPs, respectively. The remaining *mlp* genes are distributed across the genome, with approximately half located on each of the two chromosomes (Figure 2).

## 3. Flagellar Motor as an Actuator for the Chemotaxis Sensory System

### 3.1. Molecular Architecture

**Figure 4:**
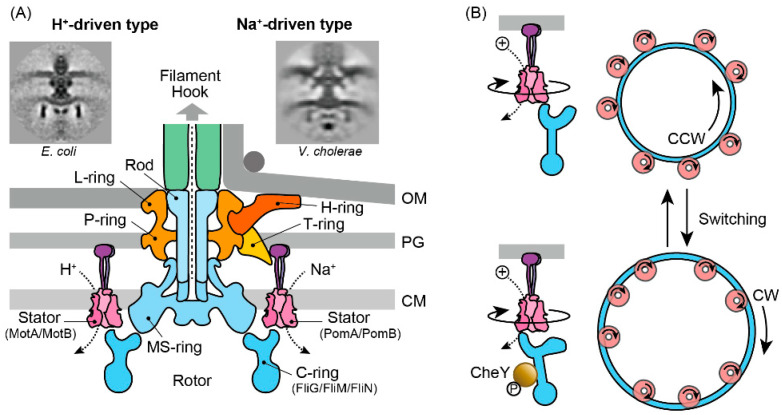
Molecular architecture of the bacterial flagellar motor. (**A**) Schematic representation of H^+^-and Na^+^-driven bacterial flagellar motors. OM, outer membrane; PG, peptidoglycan layer; CM, cytoplasmic membrane. Electron microscopy images of flagellar motors in *E. coli* and *V. cholerae* were reprinted from Chen et al. [50] with permission. (**B**) Model of motor switching between CCW and CW rotations.

The bacterial flagellum is among the most sophisticated protein complexes, comprising three primary components—the flagellar filament, the hook, and the motor (for reviews, see [47,48,49]). Here, we focus on the Na^+^- and H^+^-driven motors found in Gram-negative bacteria. Although the motor’s structure varies among species, its core architecture is conserved. For instance, the electron micrograph of the *V. cholerae* motor (Figure 4A) reveals an additional structure not seen in *E. coli*, yet the central framework remains similar [50]. At its core, the motor consists of four concentric rings, up to 45 nm in diameter, spanning the three layers of the cell envelope [51,52]. The L-ring, embedded in the outer (lipopolysaccharide) membrane, and the P-ring, located in the peptidoglycan layer, function as bushings between the rod and the cell envelope. The rod acts as a drive shaft, linking the hook to the MS-ring situated in the cytoplasmic membrane, while the C-ring, attached to the cytoplasmic face of the MS-ring, serves as the rotor. Stator complexes—composed of MotA/MotB in *E. coli* and PomA/PomB in *V. cholerae*—are anchored to the peptidoglycan layer via the peptidoglycan-binding domain of the B subunit, with up to 11 units arranged around the rotor [53,54,55]. In the *Vibrio* species, additional T- and H-rings are found in the periplasm. These structures likely reinforce the stator assembly, providing a scaffold that supports high-speed motor rotation [56,57,58].

### 3.2. Rotor–Stator Interaction

The rotational torque of the flagellar motor is generated by the interaction between the cytoplasmic site of the A subunits (MotA or PomA) within the stator complexes and the C-terminus of the FliG proteins in the C-ring [59,60]. Although the precise mechanism coupling ion transit through the stator complex to rotor rotation remains elusive, several lines of evidence indicate that Na^+^ and H^+^ motors operate via a common mechanism despite their different energy sources. First, numerous functional chimeric motors combining several components of MotA/MotB and PomA/PomB have been identified, and second, Na^+^ and H^+^ stator units can simultaneously interact with a single rotor to generate torque [61,62,63,64].

Recent advances in single-particle cryo-electron microscopy have revealed that both MotA/MotB and PomA/PomB assemble into a complex consisting of a dimer of B subunits encircled by a pentamer of A subunits, supporting the notion of a shared operational mechanism [65,66,67]. Structural and mutational analyses suggest that the A subunit undergoes sequential transitions through asymmetrically distinct states during ion transit, causing its pentameric ring to rotate around the B subunit dimer. A direct observation of the functional rotation within the stator complex, however, has yet to be achieved. This model also accounts for the motor’s ability to switch rotational direction from counterclockwise (CCW) to clockwise (CW), and vice versa (Figure 4B). Specifically, FliG in the C-ring engages with either the inner or outer segment of the pentameric A subunit ring to drive the rotor CCW or CW, respectively [67]. Moreover, cryo-electron tomography of spirochetes has revealed that the binding of phosphorylated CheY3 to FliM within the C-ring induces the structural remodeling of FliG2 [68]. In summary, the recent structural findings suggest that CheY binding alters the interaction surface between FliG and MotA. As ion conduction drives the rotation of MotA in one direction, this change directs the push on the FliG ring, enabling the motor to rotate in the desired direction.

## 4. MLPs as Sensors/Transducers for the Chemotaxis Sensory System

### 4.1. Molecular Architectures

**Figure 5:**
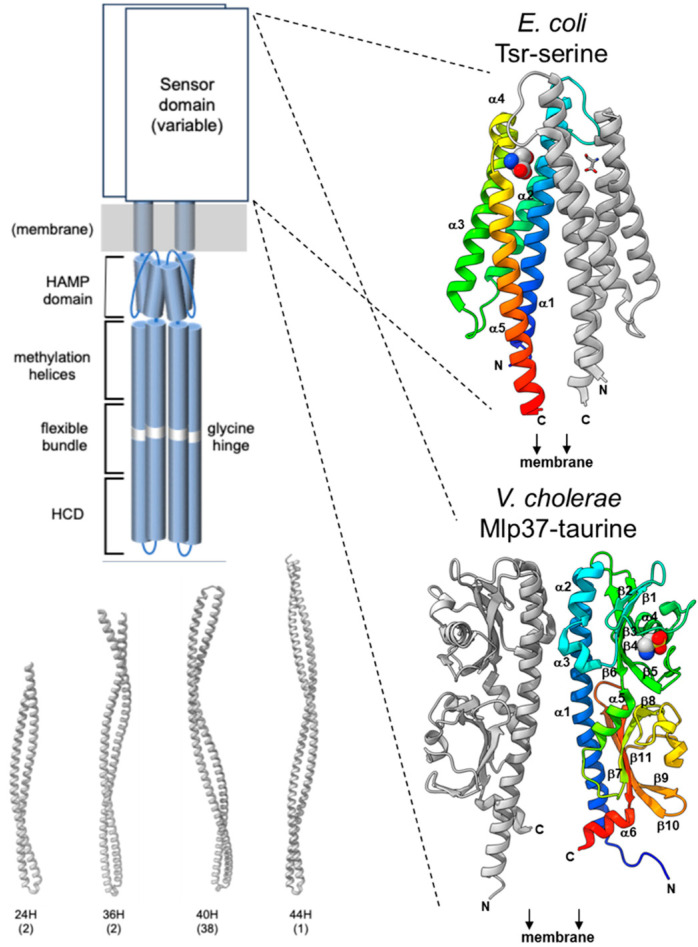
Typical structure of the chemoreceptor or MCP. Upper left panel: A schematic representation of a typical chemoreceptor homodimer. The cytoplasmic (signaling) domains are relatively conserved, comprising a HAMP domain, a flexible bundle, and highly conserved domains (HCD; see text for details). In contrast, the sensor domains are highly variable. Right panels: Representative sensor domains: The sensor domain of *E. coli* Tsr (PDB ID: 3ATP [71]) is markedly different from that of *V. cholerae* Mlp37 (PDB ID: 5AVF [72]). Lower left panel: Predicted structures of the cytoplasmic domains (beyond the HAMP domains) of classical *V. cholerae* MLPs: Mlp35 (24H, VC0395_0372), Mlp45 (36H, VC0395_0151), Mlp24 (40H, VC0395_A1741), and Mlp14 (44H, VC0395_A1015). Numbers in parentheses indicate the MLP classifications, as predicted by Alexander and Zhulin [73]. Structure predictions were performed using AlphaFold3 [74] and visualized by ChimeraX [75].

A canonical MCP, such as Tsr and Tar of *E. coli*, forms a stable homodimer—it neither forms heterodimers with related MCPs nor undergoes a monomer-to-dimer transition upon ligand binding [69,70]. Each subunit comprises two transmembrane (TM) helices along with periplasmic and cytoplasmic domains (Figure 5). In contrast, some MCP-like proteins (MLPs) lack either the periplasmic domain or the transmembrane domain. The periplasmic domain, which is flanked by the two TM helices, typically binds a specific ligand, resulting in substantial variation in its sequence and three-dimensional structure.

Conversely, the cytoplasmic domain, situated after the second TM helix, is highly conserved across species and consists of a HAMP domain, methylation helices, and a clustering/kinase regulation unit. The methylation helices usually contain multiple glutamate residues essential for adaptation; these glutamates are methylated by a methyltransferase (one of the three CheR homologs), and the methylated glutamates are subsequently demethylated by a methylesterase (one of the three CheB homologs). In some instances, the methylation sites are encoded as glutamine rather than glutamate, with the amide group being removed by a deamidase (also one of the three CheB homologs). MCPs are further classified according to the length of their methylation helices [75] (the four heptad classes are depicted in Figure 5; for a complete classification of all *V. cholerae* MLPs, see Table S1).

### 4.2. Polar Clustering

**Figure 6:**
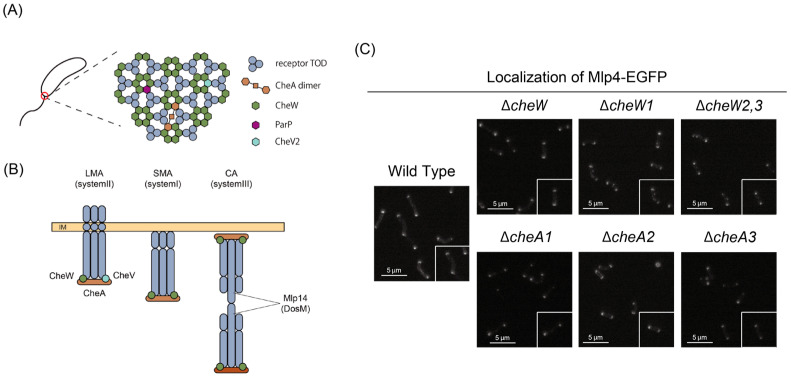
Sub-polar chemoreceptor clusters in *V. cholerae*. (**A**) The molecular organization of the System II receptor–kinase array at the flagellated pole (red circle) of *V. cholerae* cell. Cited from [39] with modification. (**B**) The *V. cholerae* cell has three distinct receptor arrays: LMA, the long membrane array corresponding to System II; SMA, the small membrane array corresponding to System III; and CA the cytoplasmic array corresponding to System I. Cited from [79] with modification. (**C**) Fluorescence microscopic observations of Mlp4-EGFP expressed in wild-type (O395N1) or its derivatives ([34]; this study) that lack either CheA or CheW homolog.

In *E. coli* and many other bacteria, MCPs assemble into an array—along with the adaptor CheW and the histidine kinase CheA—localized in sub-polar regions [76]. This receptor-kinase array is critical for facilitating highly cooperative sensing and effective adaptation [77]. Within the array, MCP trimers of dimers (TOD) are arranged in a hexagonal lattice [29,78] and interconnected by CheA and CheW [76,78]. A similar organization is observed in *V. cholerae* (Figure 6A).

Elaborate cryo-electron microscopy studies [39,79] have revealed that *V. cholerae* harbors multiple receptor–kinase arrays corresponding to its three Che systems. Specifically, components of Systems I, II, and III organize into a cytoplasmic array (CA), a long membrane array (LMA), and a short membrane array (SMA), respectively. Notably, the LMA is present under all growth conditions, whereas the CA and SMA are detectable only when cells are cultured under low oxygen and general stress conditions. The polar localization of the LMA (System II) is stringently controlled by the ParC/ParP system, with ParC acting as the key polarity determinant and ParP recruiting the array to the polar region [80]. One remarkable feature of the LMA is its lower stability compared to the well-characterized *E. coli* array, a difference that is likely due to a reduced abundance of CheA2 within the complex. This decreased stability may facilitate the incorporation of diverse MLP compositions, thereby enabling the bacterium to adapt more flexibly to environmental changes.

### 4.3. The Periplasmic Sensor Domain and Stimuli Sensed

**Figure 7:**
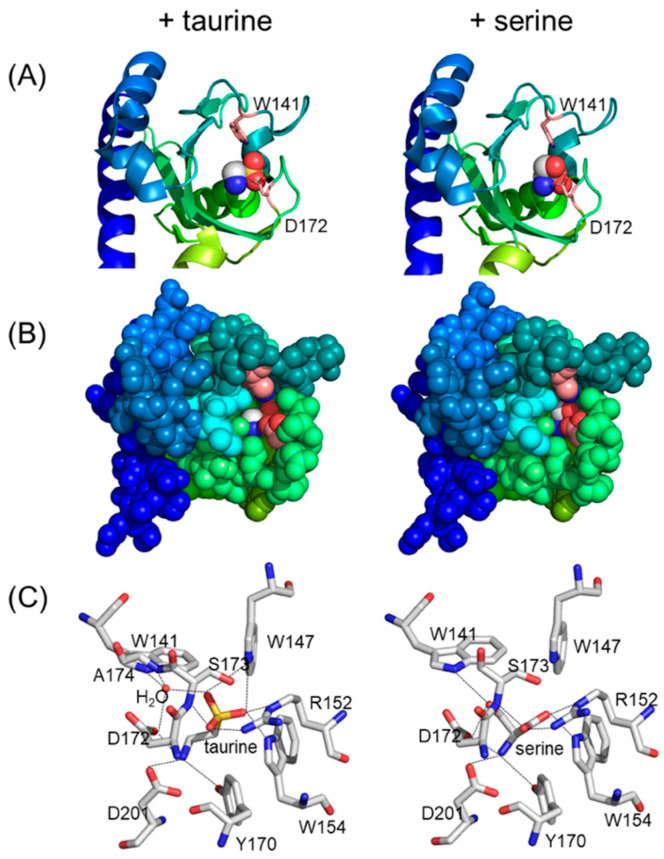
Ligand recognition by the taurine/amino acids chemoreceptor Mlp37. The ligand-binding pocket in the membrane-distal CACHE domain of Mlp37 in complex with taurine (left) and L-serine (right) are shown in ribbon representation (**A**), in ball models (**B**) painted with the same color as (**A**), and in stick models (**C**).

Several MLPs such as Mlp8, Mlp10, Mlp24, Mlp29, Mlp37, and Mlp43, harbor two tandem CACHE domains (dCACHE) [44,72], whereas others (Mlp2, Mlp3, Mlp4, Mlp9, and Mlp26) contain a single CACHE domain (sCACHE) within their periplasmic regions (Table S1). The CACHE domain is highly conserved among the periplasmic and extracellular portions of chemoreceptors from diverse Gram-positive and Gram-negative bacteria, as well as Archaea [81]. Notably, several chemoreceptors featuring dCACHE domains—such as PctA, PctB, and PctC of *Pseudomonas aeruginosa* [82,83] and McpB of *Bacillus subtilis* [84,85])—are implicated in amino acid taxis. Among the *V. cholerae* MLPs, Mlp24 and Mlp37 function as chemoreceptors for various amino acids, albeit with subtle differences in ligand specificity [44,72,86,87]; additionally, Mlp37 also recognizes taurine (2-aminoethanesulfonate) as an attractant [72,86]. Ligand binding occurs exclusively at the membrane-distal CACHE domain, and the molecular mechanisms underlying ligand recognition have been characterized at an atomic resolution (Figure 7).

Several other MLPs have been characterized or predicted based on their functions (Table S1). For instance, Mlp2 (sCACHE) is the ortholog of the VP0183 gene product from *V. parahaemolyticus*, whose sCACHE domain was co-crystallized with pyruvate [88], suggesting that Mlp2 functions as a pyruvate chemoreceptor. Similarly, the *mlp4* gene is upregulated by *N*-acetylglucosamine (GlcNAc) via the transcriptional regulator NagC [89], indicating that Mlp4 (sCACHE) likely serves as a receptor for GlcNAc or its oligomers. It is noteworthy that GlcNAc taxis may involve multiple signaling pathways, as *V. cholerae* also expresses a GlcNAc-specific PTS transporter (NagE) under NagC control, which may contribute to chemotaxis toward GlcNAc. Mlp7 (TcpI) has been implicated in pH sensing [90]. In addition, Mlp8 (AcfB; dCACHE) and the periplasmic protein AcfC—encoded by the gene co-transcribed with *mlp8*—have been reported to sense galactose-6-phosphate and mucin [91,92]. AcfC has been co-crystalized with d-malate (PDB ID: 4JB7). These reports support the idea that AcfC acts as a soluble receptor for d-malate, galactose-6-phosphate, and mucin, and that ligand-bound AcfC is recognized by Mlp8 to achieve taxis to these substances. Moreover, Mlp32, an MLP lacking a periplasmic domain but containing a cytoplasmic PAS (Per-ARNT-Sim) sensor domain, is responsible for aerotaxis and redox taxis [93]. All the above-mentioned MLPs are associated with System II. Only one MLP from another system has been functionally characterized—Mlp45 (System III) acts as an oxygen sensor. Although it lacks a periplasmic domain, it contains two tandem cytoplasmic PAS domains, each binding heme [43]; however, its signal output has been characterized only in *E. coli*.

Regarding repellent sensing in *V. cholerae*, little is known except for phenol. Phenol has been identified as a repellent in the marine bacterium *V. alginolyticus*, where it induces frequent back-and-forth movements [94]. Similar behaviors have been observed in *V. cholerae* exposed to phenol, yet the specific MCP(s) responsible for this response remain unidentified.

### 4.4. Assignment of MLPs to Three Che Systems

A comprehensive understanding of the chemotaxis and related signaling systems in *V. cholerae* requires the assignment of each MLP to one of the three Che systems. Table S1 summarizes the current attributions. With the exception of the MLPs encoded in Clusters I and III, the genomic location of an MLP does not reveal which CheA homolog (CheA1, CheA2, or CheA3) it is coupled with. Similarly, the length of an MLP’s methylation helices is not generally predictive of its coupling, except that MLPs with “36H” heptad repeats belong to System III. Most MLPs—including all those confirmed as part of System II—fall into the “40H” class, although Mlp15, encoded within the *che* gene Cluster I, also belongs to this class. Moreover, bioinformatic analyses predict that at least 17 *V. cholerae* MLPs, which share high similarity in their cytoplasmic domains with those of *V. parahaemolyticus* (a related bacterium with a single Che system), are involved in chemotaxis.

**Figure 8:**
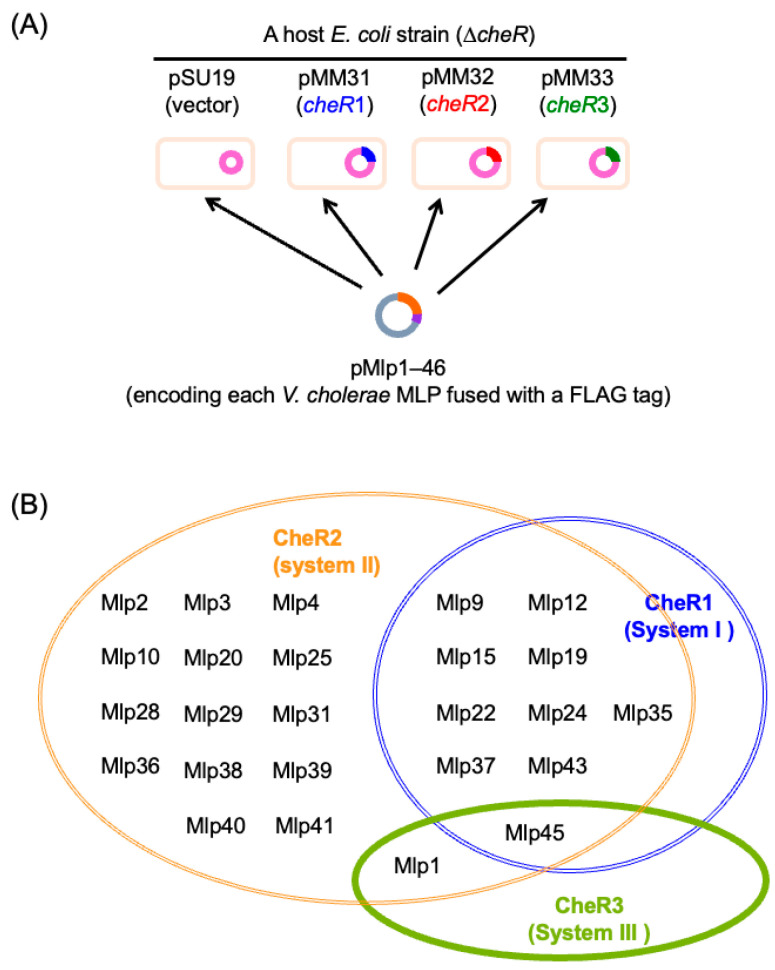
Classification of MLP by methyltransferase specificity. (**A**) Schematic diagrams of the experimental system to attribute each MLP to any of the three Che systems. Plasmids carrying each *mlp* gene were introduced into *E. coli* strains expressing each of the *cheR* genes from *V. cholerae* or none of them (harboring the plasmid pSU19, the parent vector). Substrate specificities of the CheRs were then observed to attribute each MLP to Che Systems I, II or III. (**B**) The Benn diagram summarizes attributions of MLPs in *V. cholerae* to any of the three Che systems indicated by the substrate specificity of the CheR homologs. MLPs that were not methylated by any CheR homolog are not included in the figure. For details, see Table S1.

The substrate specificity of the three methyltransferases—CheR1, CheR2, and CheR3—provides another means to assign MLPs to their respective Che systems. Our unpublished results on the methylation of individual MLPs by these CheR homologs in *E. coli* (Figure 8A) suggest that at least 25 *V. cholerae* MLPs participate in chemotaxis (Figure 8B, Figures S1 and S2, Table S1). Consistent with the cryoEM observations showing that the LMA (System II) comprises MLPs of the 40H heptad class, all the MLPs attributed to System II by this assay are of that class. Only one MLP (Mlp15) of the 40H class was attributed to System I; however, Mlp15 was also methylated by the System II methyltransferase CheR2, which aligns with the observations of Ortega et al. [79]. In contrast, Mlp1 and Mlp45 of the 36H heptad class, together with Mlp44, were assigned to System III. These System III MLPs are characterized by a distinctive C-terminal pentapeptide sequence, E-W/V-E-X-F, which may serve as a binding site for CheR3, while the C-terminal motifs for CheR1 and CheR2 binding are less apparent (Table S1). It is important to note that (i) some MLPs, such as *E. coli* Aer, were not methylated when co-expressed with any of the three CheR homologs; (ii) the cellular levels of CheR homologs were not uniform even when expressed from heterologous promoters; and (iii) the substrate specificity of the CheR homologs is relatively relaxed, as many MLPs are methylated by multiple CheR homologs, albeit with varying efficiencies (Table S1).

Another key criterion for attribution is the adaptor specificity governing polar localization. Figure 6C presents our unpublished data on the localization of Mlp4-EGFP in wild-type strains and in mutants lacking individual CheA or CheW homologs. Although the polar distribution of Mlp4-EGFP was slightly reduced in the ∆*cheA2* and ∆*cheW1* strains, the differences were minimal. For a comprehensive analysis of all MLPs, further refinements in the experimental setup—such as employing *cheV* and/or *parC/parP* mutants—will be necessary.

### 4.5. Roles in Infection and Pathogenicity

**Figure 9:**
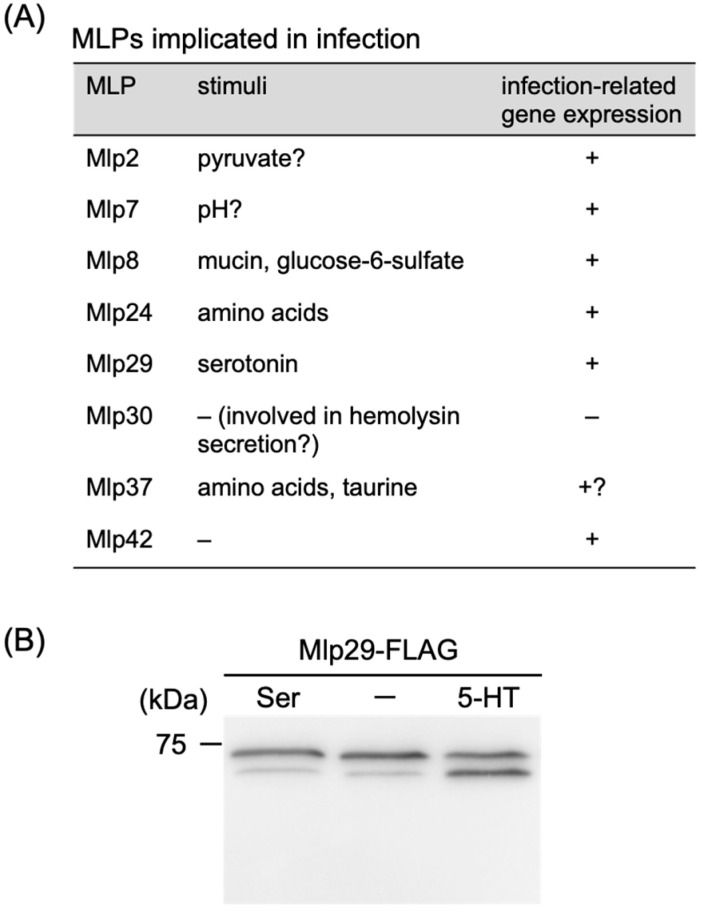
Some MLPs are implicated in infection. (**A**) Sensing stimuli and gene expression of MLPs implicated in infection. Note: +, data available; –, data not available; ?, predicted but not experimentally established. See text for detail. (**B**) Effect of serotonin (5-hydroxytryptamine) on the methylation of Mlp29. FLAG-tagged Mlp29 was examined for its methylation in the presence of serine (Ser) or serotonin (5-HT) or the absence of either of them (–) using SDS-PAGE and immunoblotting. Original images can be found in Supplementary Materials.

Several MLPs and their corresponding genes have been reported or suggested to contribute to infection and pathogenesis (Figure 9A). For example, during mouse infection, *mlp24* (also known as *mcpX*) is essential for the optimal expression of genes encoding the cholera toxin (CT) and its positive regulator ToxT [9]. Its closely related homolog, Mlp37, senses taurine—a component of bile—and chemotaxis toward taurine is enhanced at 37 °C, implying that taurine-mediated chemotaxis is particularly advantageous in human intestines [72]. Furthermore, the genes encoding Mlp7 (TcpI) and Mlp8 (AcfB) are located within the *Vibrio* Pathogenicity Island (VPI) (Figure 2), and their transcription is regulated by the master transcription factors ToxR and TcpP, which also control the expression of CT and toxin-coregulated pili (TCP). Mlp7 has been implicated in TCP formation [90], intestinal colonization [95], and motility/chemotaxis [95,96]. Mlp8, along with the periplasmic protein AcfC, has been identified as an accessory colonization factor (ACF) and is thus involved in intestinal colonization [97]. Mlp30 (HlyB) plays a role in the secretion of hemolysin [98]. Notably, several *mlp* genes—including *mlp2*, *mlp29*, and *mlp42*—are uniquely expressed during human infection [99]. Although Mlp2, an sCACHE-type transmembrane MLP predicted to sense pyruvate, has an unclear role in infection, our unpublished data indicate that Mlp29, a dCACHE-type transmembrane MLP, exhibits increased methylation upon exposure to serotonin (5-hydroxytryptamine) (Figure 9B). Given that *V. cholerae* stimulates host serotonin synthesis [100], Mlp29 is likely to play a role during infection. Interestingly, although a serotonin chemoreceptor has recently been identified in *Pseudomonas aeruginosa* [101], its periplasmic region shows relatively low similarity to that of Mlp29. Additionally, the expression of certain *mlp* genes is induced at specific stages of biofilm formation. For instance, *mlp33* is upregulated in the planktonic stage; *mlp9*, *mlp13*, *mlp15*, *mlp21*, *mlp26*, *mlp31*, *mlp36*, and *mlp44* are upregulated during the monolayer stage; and *mlp2*, *mlp3*, and *mlp22* are upregulated in the mature biofilm stage [102]. However, how these MLPs and *mlp* genes are involved in pathogenicity and biofilm formation in *V. cholerae* remains to be elucidated.

## 5. Concluding Remarks and Future Directions

The motility and chemotaxis of *Vibrio cholerae* are not only vital for its survival in diverse environments but also play a significant role in its pathogenicity. Comprehensive studies on these processes will likely aid in controlling cholera as well as other infections caused by the *Vibrio* species. Moreover, *V. cholerae* exhibits a range of intriguing biological features, including a sodium-driven flagellar motor, parallel chemotaxis-like signaling systems, and numerous unexplored environmental sensors. Compared to other bacteria with sodium-driven motility, *V. cholerae* offers the following distinct advantages: it thrives at lower salt concentrations than other *Vibrio* species and is highly amenable to molecular manipulations. Although its human pathogenicity might be a concern, the non-virulent strains—harboring mutations in the *ctxAB* genes—appear to retain wild-type motility and chemotaxis. Consequently, *V. cholerae* serves as an excellent model system for investigating sodium-driven motility and chemotaxis.

## Data Availability

Not applicable.

## Supplementary Materials

The following supporting information can be downloaded at: https://www.mdpi.com/article/10.3390/biom15030434/s1, Figure S1: MLPs methylated only by CheR2; Figure S2: MLPs methylated by (A) CheR1 and CheR2, (B) CheR2 and CheR3, and (C) all CheR species; Figure S3: The original blot image corresponding to Figure 9B; Table S1: Encoded genes, structures, functions and affiliated systems of *V. cholerae* MLPs. References [103,104,105,106] are cited in the supplementary materials.

## References

[1] Conner J.G., Teschler J.K., Jones C.J., Yildiz F.H. (2016). Staying Alive: *Vibrio cholerae*’s Cycle of Environmental Survival, Transmission, and Dissemination. Microbiol. Spectr..

[2] Reidl J., Klose K.E. (2002). *Vibrio cholerae* and cholera: Out of the water and into the host. FEMS Microbiol. Rev..

[3] Khan F., Tabassum N., Anand R., Kim Y.M. (2020). Motility of *Vibrio* spp.: Regulation and controlling strategies. Appl. Microbiol. Biotechnol..

[4] Alm R.A., Manning P.A. (1990). Characterization of the *hlyB* gene and its role in the production of the El Tor haemolysin of *Vibrio cholerae* O1. Mol. Microbiol..

[5] Banerjee R., Das S., Mukhopadhyay K., Nag S., Chakrabortty A., Chaudhuri K. (2002). Involvement of in vivo induced *cheY-4* gene of *Vibrio cholerae* in motility, early adherence to intestinal epithelial cells and regulation of virulence factors. FEBS Lett..

[6] Echazarreta M.A., Klose K.E. (2019). *Vibrio* Flagellar Synthesis. Front. Cell Infect. Microbiol..

[7] Hang L., John M., Asaduzzaman M., Bridges E.A., Vanderspurt C., Kirn T.J., Taylor R.K., Hillman J.D., Progulske-Fox A., Handfield M. (2003). Use of in vivo-induced antigen technology (IVIAT) to identify genes uniquely expressed during human infection with *Vibrio cholerae*. Proc. Natl. Acad. Sci. USA.

[8] Krukonis E.S., DiRita V.J. (2003). From motility to virulence: Sensing and responding to environmental signals in *Vibrio cholerae*. Curr. Opin. Microbiol..

[9] Lee S.H., Butler S.M., Camilli A. (2001). Selection for in vivo regulators of bacterial virulence. Proc. Natl. Acad. Sci. USA.

[10] Peterson K.M., Gellings P.S. (2018). Multiple intraintestinal signals coordinate the regulation of *Vibrio cholerae* virulence determinants. Pathog. Dis..

[11] Armitage J.P. (2024). Microbial Primer: The bacterial flagellum—How bacteria swim. Microbiology.

[12] Berg H.C. (2004). E. coli in Motion.

[13] Karmakar R. (2021). State of the art of bacterial chemotaxis. J. Basic. Microbiol..

[14] McCarter L.L. (2001). Polar flagellar motility of the *Vibrionaceae*. Microbiol. Mol. Biol. Rev..

[15] Correa N.E., Peng F., Klose K.E. (2005). Roles of the regulatory proteins FlhF and FlhG in the *Vibrio cholerae* flagellar transcription hierarchy. J. Bacteriol..

[16] Green J.C., Kahramanoglou C., Rahman A., Pender A.M., Charbonnel N., Fraser G.M. (2009). Recruitment of the earliest component of the bacterial flagellum to the old cell division pole by a membrane-associated signal recognition particle family GTP-binding protein. J. Mol. Biol..

[17] Hranitzky K.W., Mulholland A., Larson A.D., Eubanks E.R., Hart L.T. (1980). Characterization of a flagellar sheath protein of *Vibrio cholerae*. Infect. Immun..

[18] Kojima S., Yamamoto K., Kawagishi I., Homma M. (1999). The polar flagellar motor of *Vibrio cholerae* is driven by an Na^+^ motive force. J. Bacteriol..

[19] Yorimitsu T., Homma M. (2001). Na^+^-driven flagellar motor of *Vibrio*. Biochim. Biophys. Acta.

[20] Grognot M., Mittal A., Mah’moud M., Taute K.M. (2021). *Vibrio cholerae* Motility in Aquatic and Mucus-Mimicking Environments. Appl. Env. Microbiol..

[21] Xie L., Altindal T., Chattopadhyay S., Wu X.L. (2011). Bacterial flagellum as a propeller and as a rudder for efficient chemotaxis. Proc. Natl. Acad. Sci. USA.

[22] Syed K.A., Beyhan S., Correa N., Queen J., Liu J., Peng F., Satchell K.J., Yildiz F., Klose K.E. (2009). The *Vibrio cholerae* flagellar regulatory hierarchy controls expression of virulence factors. J. Bacteriol..

[23] Syed K.A., Klose K.E., Ramamurthy T., Bhattacharya S.K. (2011). Vibrio Cholerae Flagellar Synthesis and Virulence. Epidemiological and Molecular Aspects on Cholera.

[24] Yoon S.S., Mekalanos J.J. (2008). Decreased potency of the *Vibrio cholerae* sheathed flagellum to trigger host innate immunity. Infect. Immun..

[25] Dongre M., Singh B., Aung K.M., Larsson P., Miftakhova R., Persson K., Askarian F., Johannessen M., von Hofsten J., Persson J.L. (2018). Flagella-mediated secretion of a novel *Vibrio cholerae* cytotoxin affecting both vertebrate and invertebrate hosts. Commun. Biol..

[26] Buschiazzo A., Trajtenberg F. (2019). Two-Component Sensing and Regulation: How Do Histidine Kinases Talk with Response Regulators at the Molecular Level?. Annu. Rev. Microbiol..

[27] Ishii E., Eguchi Y. (2021). Diversity in Sensing and Signaling of Bacterial Sensor Histidine Kinases. Biomolecules.

[28] Parkinson J.S., Hazelbauer G.L., Falke J.J. (2015). Signaling and sensory adaptation in *Escherichia coli* chemoreceptors: 2015 update. Trends Microbiol..

[29] Briegel A., Ortega D.R., Tocheva E.I., Wuichet K., Li Z., Chen S., Muller A., Iancu C.V., Murphy G.E., Dobro M.J. (2009). Universal architecture of bacterial chemoreceptor arrays. Proc. Natl. Acad. Sci. USA.

[30] Khursigara C.M., Wu X., Zhang P., Lefman J., Subramaniam S. (2008). Role of HAMP domains in chemotaxis signaling by bacterial chemoreceptors. Proc. Natl. Acad. Sci. USA.

[31] Zhang P., Khursigara C.M., Hartnell L.M., Subramaniam S. (2007). Direct visualization of *Escherichia coli* chemotaxis receptor arrays using cryo-electron microscopy. Proc. Natl. Acad. Sci. USA.

[32] Zhang P., Weis R.M., Peters P.J., Subramaniam S. (2007). Electron tomography of bacterial chemotaxis receptor assemblies. Methods Cell Biol..

[33] Gosink K.K., Kobayashi R., Kawagishi I., Hase C.C. (2002). Analyses of the roles of the three *cheA* homologs in chemotaxis of *Vibrio cholerae*. J. Bacteriol..

[34] Heidelberg J.F., Eisen J.A., Nelson W.C., Clayton R.A., Gwinn M.L., Dodson R.J., Haft D.H., Hickey E.K., Peterson J.D., Umayam L. (2000). DNA sequence of both chromosomes of the cholera pathogen *Vibrio cholerae*. Nature.

[35] Hess J.F., Oosawa K., Kaplan N., Simon M.I. (1988). Phosphorylation of three proteins in the signaling pathway of bacterial chemotaxis. Cell.

[36] Kuo S.C., Koshland D.E. (1987). Roles of *cheY* and *cheZ* gene products in controlling flagellar rotation in bacterial chemotaxis of *Escherichia coli*. J. Bacteriol..

[37] Kristich C.J., Ordal G.W. (2002). *Bacillus subtilis* CheD is a chemoreceptor modification enzyme required for chemotaxis. J. Biol. Chem..

[38] Alexander R.P., Lowenthal A.C., Harshey R.M., Ottemann K.M. (2010). CheV: CheW-like coupling proteins at the core of the chemotaxis signaling network. Trends Microbiol..

[39] Yang W., Alvarado A., Glatter T., Ringgaard S., Briegel A. (2018). Baseplate variability of *Vibrio cholerae* chemoreceptor arrays. Proc. Natl. Acad. Sci. USA.

[40] Hiremath G., Nishiyama S., Kawagishi I. (2013). CheV1 plays an important role in chemotaxis of *Vibrio cholerae*. Int. J. Biosci. Biochem. Bioinform..

[41] Hyakutake A., Homma M., Austin M.J., Boin M.A., Häse C.C., Kawagishi I. (2005). Only one of the five CheY homologs in *Vibrio cholerae* directly switches flagellar rotation. J. Bacteriol..

[42] Hiremath G., Hyakutake A., Yamamoto K., Ebisawa T., Nakamura T., Nishiyama S., Homma M., Kawagishi I. (2015). Hypoxia-induced localization of chemotaxis-related signaling proteins in *Vibrio cholerae*. Mol. Microbiol..

[43] Greer-Phillips S.E., Sukomon N., Chua T.K., Johnson M.S., Crane B.R., Watts K.J. (2018). The Aer2 receptor from *Vibrio cholerae* is a dual PAS-heme oxygen sensor. Mol. Microbiol..

[44] Nishiyama S., Suzuki D., Itoh Y., Suzuki K., Tajima H., Hyakutake A., Homma M., Butler-Wu S.M., Camilli A., Kawagishi I. (2012). Mlp24 (McpX) of *Vibrio cholerae* implicated in pathogenicity functions as a chemoreceptor for multiple amino acids. Infect. Immun..

[45] Le Moual H., Koshland D.E. (1996). Molecular evolution of the C-terminal cytoplasmic domain of a superfamily of bacterial receptors involved in taxis. J. Mol. Biol..

[46] Zhulin I.B. (2001). The superfamily of chemotaxis transducers: From physiology to genomics and back. Adv. Microb. Physiol..

[47] Berg H.C. (2003). The rotary motor of bacterial flagella. Annu. Rev. Biochem..

[48] Nakamura S., Minamino T. (2019). Flagella-Driven Motility of Bacteria. Biomolecules.

[49] Sowa Y., Berry R.M. (2008). Bacterial flagellar motor. Q. Rev. Biophys..

[50] Chen S., Beeby M., Murphy G.E., Leadbetter J.R., Hendrixson D.R., Briegel A., Li Z., Shi J., Tocheva E.I., Muller A. (2011). Structural diversity of bacterial flagellar motors. EMBO J..

[51] Tan J., Zhang X., Wang X., Xu C., Chang S., Wu H., Wang T., Liang H., Gao H., Zhou Y. (2021). Structural basis of assembly and torque transmission of the bacterial flagellar motor. Cell.

[52] Thomas D.R., Morgan D.G., DeRosier D.J. (1999). Rotational symmetry of the C ring and a mechanism for the flagellar rotary motor. Proc. Natl. Acad. Sci. USA.

[53] Kojima S., Imada K., Sakuma M., Sudo Y., Kojima C., Minamino T., Homma M., Namba K. (2009). Stator assembly and activation mechanism of the flagellar motor by the periplasmic region of MotB. Mol. Microbiol..

[54] Leake M.C., Chandler J.H., Wadhams G.H., Bai F., Berry R.M., Armitage J.P. (2006). Stoichiometry and turnover in single, functioning membrane protein complexes. Nature.

[55] Reid S.W., Leake M.C., Chandler J.H., Lo C.J., Armitage J.P., Berry R.M. (2006). The maximum number of torque-generating units in the flagellar motor of *Escherichia coli* is at least 11. Proc. Natl. Acad. Sci. USA.

[56] Terashima H., Fukuoka H., Yakushi T., Kojima S., Homma M. (2006). The *Vibrio* motor proteins, MotX and MotY, are associated with the basal body of Na-driven flagella and required for stator formation. Mol. Microbiol..

[57] Terashima H., Li N., Sakuma M., Koike M., Kojima S., Homma M., Imada K. (2013). Insight into the assembly mechanism in the supramolecular rings of the sodium-driven *Vibrio* flagellar motor from the structure of FlgT. Proc. Natl. Acad. Sci. USA.

[58] Zhu S., Nishikino T., Takekawa N., Terashima H., Kojima S., Imada K., Homma M., Liu J. (2020). In Situ Structure of the *Vibrio* Polar Flagellum Reveals a Distinct Outer Membrane Complex and Its Specific Interaction with the Stator. J. Bacteriol..

[59] Lloyd S.A., Whitby F.G., Blair D.F., Hill C.P. (1999). Structure of the C-terminal domain of FliG, a component of the rotor in the bacterial flagellar motor. Nature.

[60] Zhou J., Lloyd S.A., Blair D.F. (1998). Electrostatic interactions between rotor and stator in the bacterial flagellar motor. Proc. Natl. Acad. Sci. USA.

[61] Asai Y., Yakushi T., Kawagishi I., Homma M. (2003). Ion-coupling determinants of Na+-driven and H+-driven flagellar motors. J. Mol. Biol..

[62] Gosink K.K., Häse C.C. (2000). Requirements for conversion of the Na^+^-driven flagellar motor of *Vibrio cholerae* to the H^+^-driven motor of *Escherichia coli*. J. Bacteriol..

[63] Paulick A., Delalez N.J., Brenzinger S., Steel B.C., Berry R.M., Armitage J.P., Thormann K.M. (2015). Dual stator dynamics in the *Shewanella oneidensis* MR-1 flagellar motor. Mol. Microbiol..

[64] Sowa Y., Homma M., Ishijima A., Berry R.M. (2014). Hybrid-fuel bacterial flagellar motors in *Escherichia coli*. Proc. Natl. Acad. Sci. USA.

[65] Deme J.C., Johnson S., Vickery O., Aron A., Monkhouse H., Griffiths T., James R.H., Berks B.C., Coulton J.W., Stansfeld P.J. (2020). Structures of the stator complex that drives rotation of the bacterial flagellum. Nat. Microbiol..

[66] Hu H., Popp P.F., Santiveri M., Roa-Eguiara A., Yan Y., Martin F.J.O., Liu Z., Wadhwa N., Wang Y., Erhardt M. (2023). Ion selectivity and rotor coupling of the *Vibrio* flagellar sodium-driven stator unit. Nat. Commun..

[67] Santiveri M., Roa-Eguiara A., Kuhne C., Wadhwa N., Hu H., Berg H.C., Erhardt M., Taylor N.M.I. (2020). Structure and Function of Stator Units of the Bacterial Flagellar Motor. Cell.

[68] Chang Y., Zhang K., Carroll B.L., Zhao X., Charon N.W., Norris S.J., Motaleb M.A., Li C., Liu J. (2020). Molecular mechanism for rotational switching of the bacterial flagellar motor. Nat. Struct. Mol. Biol..

[69] Falke J.J., Koshland D.E. (1987). Global flexibility in a sensory receptor: A site-directed cross-linking approach. Science.

[70] Milligan D.L., Koshland D.E. (1988). Site-directed cross-linking. Establishing the dimeric structure of the aspartate receptor of bacterial chemotaxis. J. Biol. Chem..

[71] Tajima H., Imada K., Sakuma M., Hattori F., Nara T., Kamo N., Homma M., Kawagishi I. (2011). Ligand specificity determined by differentially arranged common ligand-binding residues in bacterial amino acid chemoreceptors Tsr and Tar. J. Biol. Chem..

[72] Nishiyama S., Takahashi Y., Yamamoto K., Suzuki D., Itoh Y., Sumita K., Uchida Y., Homma M., Imada K., Kawagishi I. (2016). Identification of a *Vibrio cholerae* chemoreceptor that senses taurine and amino acids as attractants. Sci. Rep..

[73] Alexander R.P., Zhulin I.B. (2007). Evolutionary genomics reveals conserved structural determinants of signaling and adaptation in microbial chemoreceptors. Proc. Natl. Acad. Sci. USA.

[74] Abramson J., Adler J., Dunger J., Evans R., Green T., Pritzel A., Ronneberger O., Willmore L., Ballard A.J., Bambrick J. (2024). Accurate structure prediction of biomolecular interactions with AlphaFold 3. Nature.

[75] Meng E.C., Goddard T.D., Pettersen E.F., Couch G.S., Pearson Z.J., Morris J.H., Ferrin T.E. (2023). UCSF ChimeraX: Tools for structure building and analysis. Protein Sci..

[76] Liu J., Hu B., Morado D.R., Jani S., Manson M.D., Margolin W. (2012). Molecular architecture of chemoreceptor arrays revealed by cryoelectron tomography of *Escherichia coli* minicells. Proc. Natl. Acad. Sci. USA.

[77] Gestwicki J.E., Kiessling L.L. (2002). Inter-receptor communication through arrays of bacterial chemoreceptors. Nature.

[78] Briegel A., Li X., Bilwes A.M., Hughes K.T., Jensen G.J., Crane B.R. (2012). Bacterial chemoreceptor arrays are hexagonally packed trimers of receptor dimers networked by rings of kinase and coupling proteins. Proc. Natl. Acad. Sci. USA.

[79] Ortega D.R., Kjaer A., Briegel A. (2020). The chemosensory systems of *Vibrio cholerae*. Mol. Microbiol..

[80] Ringgaard S., Schirner K., Davis B.M., Waldor M.K. (2011). A family of ParA-like ATPases promotes cell pole maturation by facilitating polar localization of chemotaxis proteins. Genes. Dev..

[81] Anantharaman V., Aravind L. (2000). Cache—A signaling domain common to animal Ca^2+^-channel subunits and a class of prokaryotic chemotaxis receptors. Trends Biochem. Sci..

[82] Kuroda A., Kumano T., Taguchi K., Nikata T., Kato J., Ohtake H. (1995). Molecular cloning and characterization of a chemotactic transducer gene in Pseudomonas aeruginosa. J. Bacteriol..

[83] Taguchi K., Fukutomi H., Kuroda A., Kato J., Ohtake H. (1997). Genetic identification of chemotactic transducers for amino acids in *Pseudomonas aeruginosa*. Microbiology.

[84] Hanlon D.W., Ordal G.W. (1994). Cloning and characterization of genes encoding methyl-accepting chemotaxis proteins in *Bacillus subtilis*. J. Biol. Chem..

[85] Muller J., Schiel S., Ordal G.W., Saxild H.H. (1997). Functional and genetic characterization of *mcpC*, which encodes a third methyl-accepting chemotaxis protein in *Bacillus subtilis*. Microbiology.

[86] Takahashi Y., Nishiyama S., Kawagishi I., Imada K. (2020). Structural basis of the binding affinity of chemoreceptors Mlp24p and Mlp37p for various amino acids. Biochem. Biophys. Res. Commun..

[87] Takahashi Y., Nishiyama S., Sumita K., Kawagishi I., Imada K. (2019). Calcium Ions Modulate Amino Acid Sensing of the Chemoreceptor Mlp24 of *Vibrio cholerae*. J. Bacteriol..

[88] Goers Sweeney E., Henderson J.N., Goers J., Wreden C., Hicks K.G., Foster J.K., Parthasarathy R., Remington S.J., Guillemin K. (2012). Structure and proposed mechanism for the pH-sensing *Helicobacter pylori* chemoreceptor TlpB. Structure.

[89] Ghosh S., Rao K.H., Sengupta M., Bhattacharya S.K., Datta A. (2011). Two gene clusters co-ordinate for a functional N-acetylglucosamine catabolic pathway in *Vibrio cholerae*. Mol. Microbiol..

[90] Taylor R.K., Manoil C., Mekalanos J.J. (1989). Broad-host-range vectors for delivery of TnphoA: Use in genetic analysis of secreted virulence determinants of *Vibrio cholerae*. J. Bacteriol..

[91] Selvaraj P., Gupta R., Peterson K.M. (2015). The *Vibrio cholerae* ToxR Regulon Encodes Host-Specific Chemotaxis Proteins that Function in Intestinal Colonization. SOJ Microbiol. Infect. Dis..

[92] Valiente E., Davies C., Mills D.C., Getino M., Ritchie J.M., Wren B.W. (2018). *Vibrio cholerae* accessory colonisation factor AcfC: A chemotactic protein with a role in hyperinfectivity. Sci. Rep..

[93] Boin M.A., Häse C.C. (2007). Characterization of *Vibrio cholerae* aerotaxis. FEMS Microbiol. Lett..

[94] Homma M., Oota H., Kojima S., Kawagishi I., Imae Y. (1996). Chemotactic responses to an attractant and a repellent by the polar and lateral flagellar systems of *Vibrio alginolyticus*. Microbiology.

[95] Chaparro A.P., Ali S.K., Klose K.E. (2010). The ToxT-dependent methyl-accepting chemoreceptors AcfB and TcpI contribute to *Vibrio cholerae* intestinal colonization. FEMS Microbiol. Lett..

[96] Harkey C.W., Everiss K.D., Peterson K.M. (1994). The *Vibrio cholerae* toxin-coregulated-pilus gene *tcpI* encodes a homolog of methyl-accepting chemotaxis proteins. Infect. Immun..

[97] Everiss K.D., Hughes K.J., Kovach M.E., Peterson K.M. (1994). The *Vibrio cholerae acfB* colonization determinant encodes an inner membrane protein that is related to a family of signal-transducing proteins. Infect. Immun..

[98] Jeffery C.J., Koshland D.E. (1993). *Vibrio cholerae hlyB* is a member of the chemotaxis receptor gene family. Protein Sci..

[99] Rollins S.M., Peppercorn A., Hang L., Hillman J.D., Calderwood S.B., Handfield M., Ryan E.T. (2005). In vivo induced antigen technology (IVIAT). Cell Microbiol..

[100] Jugder B.E., Batista J.H., Gibson J.A., Cunningham P.M., Asara J.M., Watnick P.I. (2022). *Vibrio cholerae* high cell density quorum sensing activates the host intestinal innate immune response. Cell Rep..

[101] Monteagudo-Cascales E., Lozano-Montoya A., Krell T. (2024). *Pseudomonas aeruginosa* performs chemotaxis to serotonin, dopamine, epinephrine, and norepinephrine. bioRxiv.

[102] Moorthy S., Watnick P.I. (2005). Identification of novel stage-specific genetic requirements through whole genome transcription profiling of *Vibrio cholerae* biofilm development. Mol. Microbiol..

[103] Murphy S.G., Johnson B.A., Ledoux C.M., Dorr T. (2021). *Vibrio cholerae*'s mysterious Seventh Pandemic island (VSP-II) encodes novel Zur-regulated zinc starvation genes involved in chemotaxis and cell congregation. PLoS Genet.

[104] Irazoki O., Ter Beek J., Alvarez L., Mateus A., Colin R., Typas A., Savitski M.M., Sourjik V., Berntsson R.P., Cava F. (2023). D-amino acids signal a stress-dependent run-away response in *Vibrio cholerae*. Nat. Microbiol..

[105] Briegel A., Ortega D.R., Mann P., Kjaer A., Ringgaard S., Jensen G.J. (2016). Chemotaxis cluster 1 proteins form cytoplasmic arrays in *Vibrio cholerae* and are stabilized by a double signaling domain receptor DosM. Proc. Natl. Acad. Sci. USA.

[106] Shiomi D., Zhulin I.B., Homma M., Kawagishi I. (2002). Dual recognition of the bacterial chemoreceptor by chemotaxis-specific domains of the CheR methyltransferase. J. Biol. Chem..

