# Supplementary material for: Chemotaxis and Related Signaling Systems in Vibrio cholerae
Source: Biomolecules. 2025 Mar 18;15(3):434. doi: 10.3390/biom15030434 (PMC11940527; doi:10.3390/biom15030434)
Supplement: Supplementary file 1 [file biomolecules-15-00434-s001.zip › biomolecules-3431565_FigS1-S3_Legends_revised.pdf]

**Legends to supplemental figures**

**Figure S1.** MLPs methylated only by CheR2. Lanes in which MLP methylation was detected are marked by dotted boxes. See text for detail. Lanes: v, without any CheR (vector); R1, co-expressed with CheR1; R2, co-expressed with CheR2; R3, co-expressed with CheR3.

**Figure S2.** MLPs methylated by (A) CheR1 and CheR2, (B) CheR2 and CheR3, and (C) all CheR species. Lanes in which MLP methylation was detected are marked by dotted boxes. See text for detail. Lanes are labeled as described in the legend to Figure S1.

**Figure S3.** The original blot image corresponding to Figure 9B. Lanes 6 to 8, located immediately to the right of lane 5 (blank), were extracted and used in Figure 9B.
