# Supplementary material for: Chemotaxis and Related Signaling Systems in Vibrio cholerae
Source: Biomolecules. 2025 Mar 18;15(3):434. doi: 10.3390/biom15030434 (PMC11940527; doi:10.3390/biom15030434)
Supplement: Supplementary file 1 [file biomolecules-15-00434-s001.zip › biomolecules-3431565_FigS1-S3_revised.pdf]

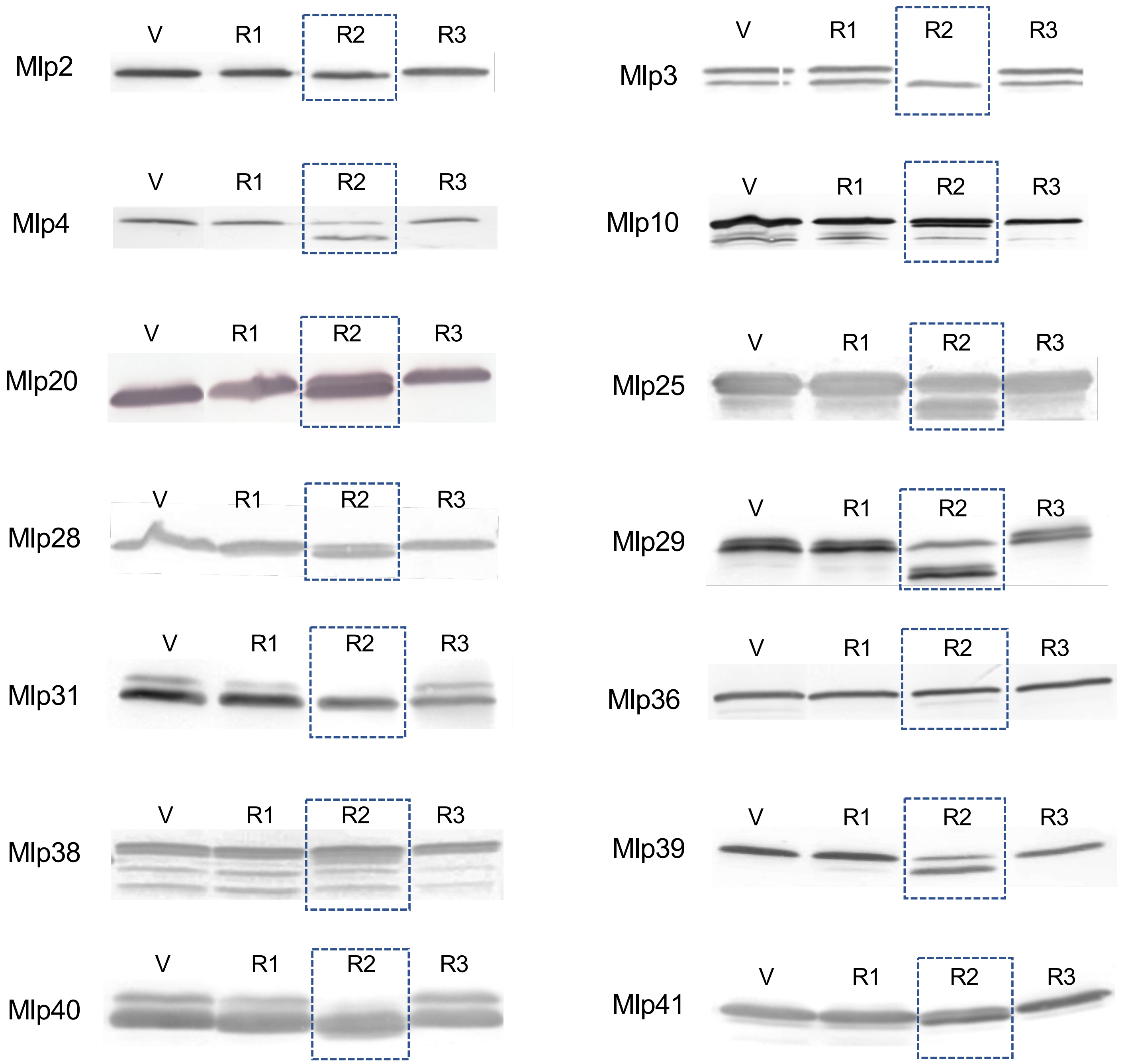

Figure S1 MLPs methylated only by CheR2

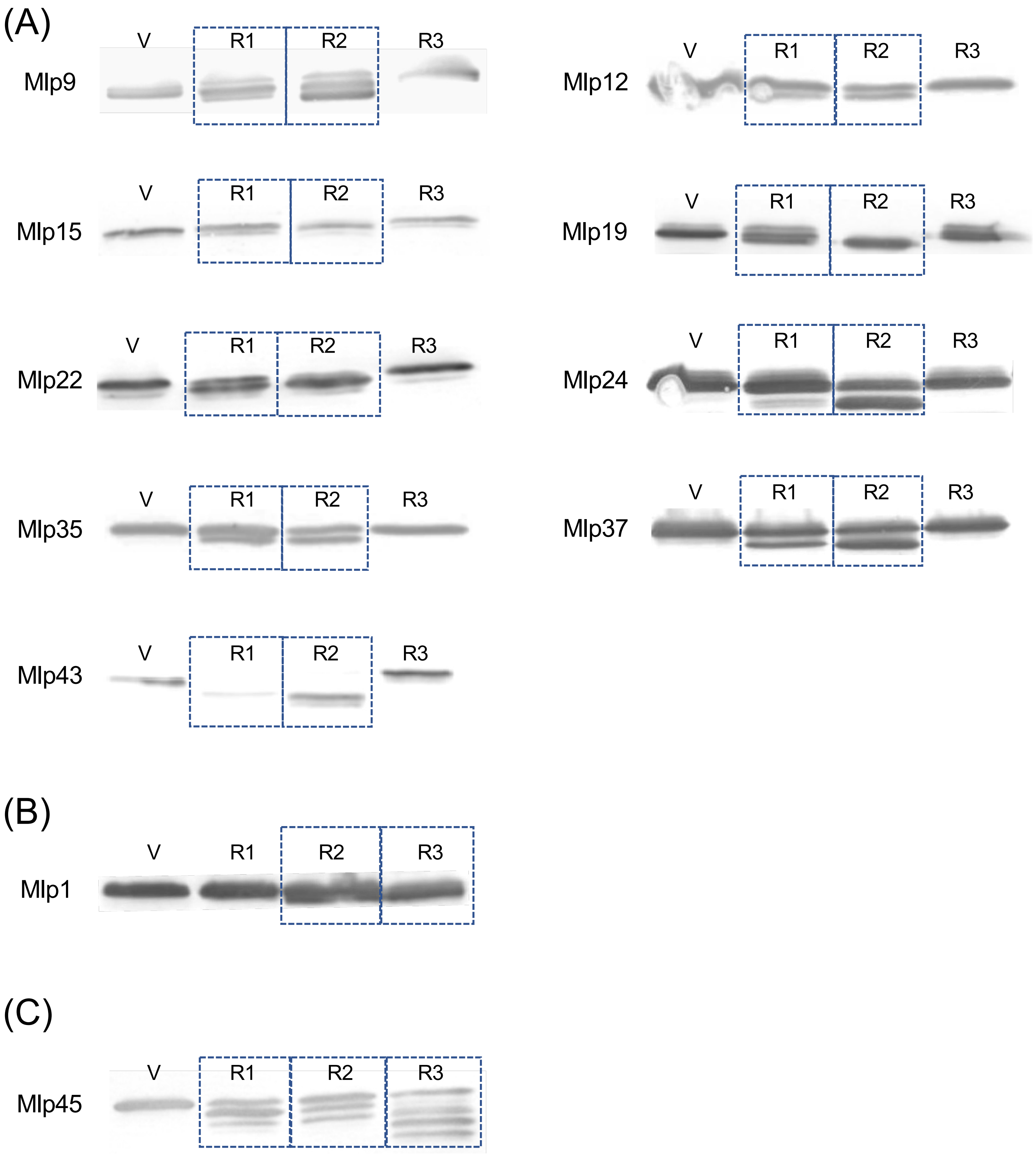

Figure S2 MLPs methylated by various CheRs

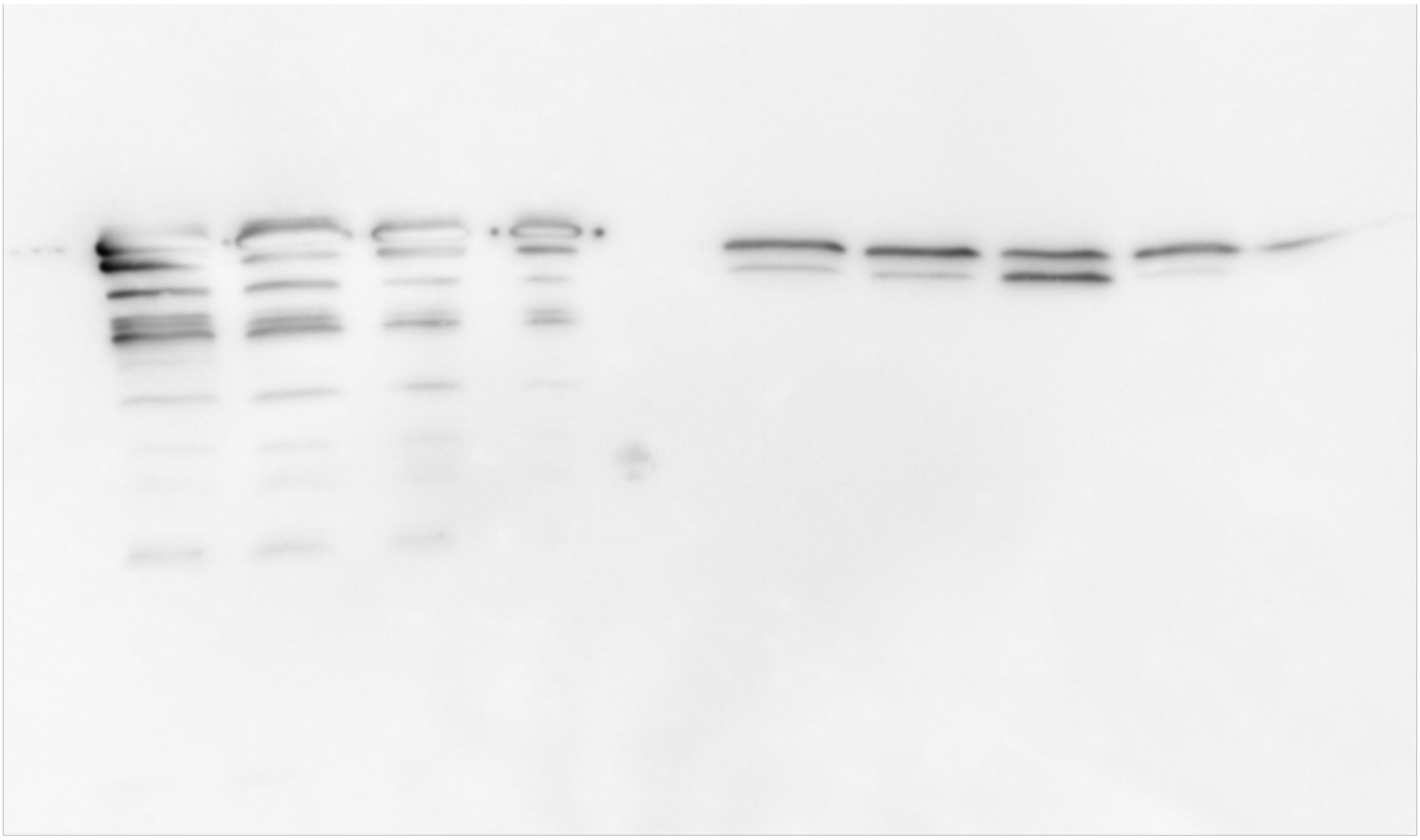

Figure S3 The original blot image corresponding to Figure 9B
