# Supplementary material for: Chemotaxis and Related Signaling Systems in Vibrio cholerae
Source: Biomolecules. 2025 Mar 18;15(3):434. doi: 10.3390/biom15030434 (PMC11940527; doi:10.3390/biom15030434)
Supplement: Supplementary file 1 [file biomolecules-15-00434-s001.zip › biomolecules-3431565_TableS1_revised.pdf]

Table S1. Encoded genes, structures, functions and affiliated systems of *V. cholerae* MLPs.

MLP	synonym	El Tor (vch)	Classical (vco)	Classical (vcr)	peri[1)]	function[2)]	heptad[3)]	C-termini[4)]	methylation[5)]	system[6)]	references
Mlp01		vc0098	VC0395_A2418	VC395_0082			36H	EWESF	R2/R3	III	
Mlp02		vc0216	VC0395_A2596	VC395_0248	sCache	pyruvate taxis? involved in infection?	40H	GHFKL	R2	II	
Mlp03		vc0282	VC0395_A2659	VC395_0311	sCache		40H	AVFKI	R2	II	
Mlp04		vc0449	VC0395_A2871	VC395_0493	sCache	GlcNAc taxis?	40H	FEVTR	R2	II	
Mlp05	Aer-1, AerB	vc0512	–	–	cPAS	negative aerotaxis	40H	WERNK	NT	II	[1,2]
Mlp06		vc0514	–	–	dCache		40H	NEFKI	NT	II?	
Mlp07	TcpI	vc0825	VC0395_A0350	VC395_0841		pH sensing?, involved in infection?	40H	IELTS	NT	II	[3]
Mlp08	AcfB	vc0840	VC0395_A0365	VC395_0856	dCache	mucin taxis, glucose-6-sulfate taxis, involved in infection?	40H	SKFII	NT	II	[4]
Mlp09		vc1248	VC0395_A0868	VC395_1367	sCache		40H	TAFKL	R1/R2	II	
Mlp10		vc1289	VC0395_A0908	VC395_1408	dCache		40H	RRFKL	R2	II	
Mlp11		vc1298	VC0395_A0916	VC395_1417			40H	ARKPH	NM	II?	
Mlp12	MCP_{DRK}	vc1313	VC0395_A0931	VC395_1432			40H	RFKIR	R1/R2	II	[5]
Mlp13		vc1394	VC0395_A1006	VC395_1513			UM	KIELF	NM	I	
Mlp14	DosM	vc1403	VC0395_A1015	VC395_1522		involved in clustering of cytoplasmic MLPs	44H	ELQSI	NM	I	[6]
Mlp15		vc1405	VC0395_A1016	VC395_1524			40H	RFVIS	R1/R2	I	
Mlp16		vc1406	VC0395_A1017	VC395_1525			24H	VSNIV	NM	I	
Mlp17		vc1413	VC0395_A1023	VC395_1532			40H	RFRVQ	NM	II?	
Mlp18		vc1535	VC0395_A1141	VC395_1653			40H	KYELN	NM	II?	
Mlp19		vc1643	VC0395_A1249	VC395_1760			40H	ARYNA	R1/R2	II	
Mlp20		vc1859	VC0395_A1450	VC395_1974			40H	QFKVN	R2	II	
Mlp21		vc1868	VC0395_A1459	VC395_1983			40H	RCFTL	NT	II?	
Mlp22		vc1898	VC0395_A1488	VC395_2013			40H	HRFKL	R1/R2	II	
Mlp23		vc1967	VC0395_A1554	VC395_2082			40H	SKFKI	NM	II?	
Mlp24	McpX	vc2161	VC0395_A1741	VC395_2275	dCache	taxis to AAs, involved in infection?	40H	NTFIV	R1/R2	II	[7-9]
Mlp25		vc2439	VC0395_A2016	VC395_2554			40H	TAFRV	R2	II	
Mlp26		vca0008	VC0395_0122	VC395_A0008	sCache		40H	ARFKV	NM	II?	
Mlp27		vca0031	VC0395_0103	VC395_A0027			40H	KQLSH	NM	II?	
Mlp28		vca0068	VC0395_0070	VC395_A0061			40H	QYFRV	R2	II	
Mlp29		vca0176	VC0395_1102	VC395_A0167	dCache	serotonin taxis, involved in infection?	40H	GNFKL	R2	II	
Mlp30	HlyB	vca0220	VC0395_1007	VC395_A0257		involved in hemolysin secretion?	40H	GSFEL	NM	II?	[10]
Mlp31		vca0268	VC0395_0962	VC395_A0305			40H	KFKLR	R2	II?	

Mlp32	Aer-2	vca0658	VC0395_0602	VC395_A0654	cPAS	aerotaxis	40H	QFAQK	NM	II	[1]
Mlp33		vca0663	VC0395_0606	VC395_A0649			40H	FRTNRD	NM	II?	
Mlp34		vca0773	VC0395_0713	VC395_A0541			40H	ARYRT	NT	II?	
Mlp35		vca0864	VC0395_0372	VC395_A0888			24H	VSQLK	R1/R2	I	
Mlp36		vca0906	VC0395_0331	VC395_A0931			40H	AHIRT	R2	II	
Mlp37		vca0923	VC0395_0316	VC395_A0948	dCache	taxis to AAs and taurine, involved in infection?	40H	NKFKV	R1/R2	II	[11]
Mlp38		vca0974	VC0395_0265	VC395_A0999			40H	SFFKL	R2	II	
Mlp39		vca0979	VC0395_0259	VC395_A1004			40H	AKFRT	R2	II	
Mlp40	Aer-3	vca0988	VC0395_0250	VC395_A1012	cPAS		40H	QFRRV	R2	II	[1]
Mlp41		vca1034	VC0395_0207	VC395_A1057			40H	AHFKV	R2	II	
Mlp42		vca1056	VC0395_0185	VC395_A1079		involved in infection?	40H	GQFRY	NT	II?	
Mlp43		vca1069	VC0395_0173	VC395_A1091	dCache		40H	SRFKL	R1/R2	II	
Mlp44		vca1088	VC0395_0155	VC395_A1109			UM	EVELF	NM	III	
Mlp45	Aer2	vca1092	VC0395_0151	VC395_A1113		aerotaxis	36H	EWEEF	R1/R2/R3	III	[12]
Mlp46		–	VC0395_0210	VC395_A1054			40H	QRFRL	NM	II?	

Notes:

1). This column shows the domain architectures of MLPs. "sCache" and "dCache" mean the MLP possesses single and double Cache domain(s) , respectively, in its periplasmic region. See text for detail. "cPAS" means the MLP possesses PAS domain in its cytoplasmic domain.

2). This column represents established or deduced (marked with "?") function(s) of each MLP. Abbreviations: AAs, amino acids; GlcANc, *N*-acetyl-D-glucosamine.

3). Numbers of heptad repeats (H) of its cytoplasmic domain determined by Alexander and Zhulin (2001) [13] are shown. "UM" means "Unaligned Membrane-bound".

4). The C-terminal five amino acid (pentapeptide) sequences of each MLP are shown. Characteristic pentapeptide sequences at their C-terminus, E-W/V-E-X-F, were seen in Che system III MLPs, Mlp1 and Mlp45. These sequences are very similar to those appear in *E. coli* MCPs (NWETF) which provide binding site for CheR [14]. See text for detail.

5). Summary of the MLPs methylation by a two-plasmid co-expression experiments. R1, R2 and R3 mean the detection of methylation of the MLP by CheR1, CheR2 and CheR3, respectively. NT, not tested; NM, no detectable methylation observed by any of CheR1-CheR3. See text, as well as Figures 8, S1 and S2 for detail.

6). Attributions of each MLP to Che systems. Roman numbers (I~III) mean the corresponding MLP is deduced to belong primarily to Che system I, II, III, respectively. Question marks "?" mean that the Che system attributions of the MLPs were only determined by number of its heptad repeat or genomic architecture, but not confirmed by the previous studies nor our methylation assays. See text for detail.

References (Table S1)

1. Boin, M.A.; Häse, C.C. Characterization of *Vibrio cholerae* aerotaxis. *FEMS Microbiol Lett* **2007**, *276*, 193-201, doi:10.1111/j.1574-6968.2007.00931.x.
2. Murphy, S.G.; Johnson, B.A.; Ledoux, C.M.; Dorr, T. *Vibrio cholerae*'s mysterious Seventh Pandemic island (VSP-II) encodes novel Zur-regulated zinc starvation genes involved in chemotaxis and cell congregation. *PLoS Genet* **2021**, *17*, e1009624, doi:10.1371/journal.pgen.1009624.
3. Harkey, C.W.; Everiss, K.D.; Peterson, K.M. The *Vibrio cholerae* toxin-coregulated-pilus gene *tcpI* encodes a homolog of methyl-accepting chemotaxis proteins. *Infect Immun* **1994**, *62*, 2669-2678, doi:10.1128/iai.62.7.2669-2678.1994.
4. Everiss, K.D.; Hughes, K.J.; Kovach, M.E.; Peterson, K.M. The *Vibrio cholerae acfB* colonization determinant encodes an inner membrane protein that is related to a family of signal-transducing proteins. *Infect Immun* **1994**, *62*, 3289-3298, doi:10.1128/iai.62.8.3289-3298.1994.
5. Irazoki, O.; Ter Beek, J.; Alvarez, L.; Mateus, A.; Colin, R.; Typas, A.; Savitski, M.M.; Sourjik, V.; Berntsson, R.P.; Cava, F. D-amino acids signal a stress-dependent run-away response in *Vibrio cholerae*. *Nat Microbiol* **2023**, *8*, 1549-1560, doi:10.1038/s41564-023-01419-6.
6. Briegel, A.; Ortega, D.R.; Mann, P.; Kjaer, A.; Ringgaard, S.; Jensen, G.J. Chemotaxis cluster 1 proteins form cytoplasmic arrays in *Vibrio cholerae* and are stabilized by a double signaling domain receptor DosM. *Proc Natl Acad Sci U S A* **2016**, *113*, 10412-10417, doi:10.1073/pnas.1604693113.
7. Lee, S.H.; Butler, S.M.; Camilli, A. Selection for *in vivo* regulators of bacterial virulence. *Proc Natl Acad Sci U S A* **2001**, *98*, 6889-6894, doi:10.1073/pnas.111581598.
8. Nishiyama, S.; Suzuki, D.; Itoh, Y.; Suzuki, K.; Tajima, H.; Hyakutake, A.; Homma, M.; Butler-Wu, S.M.; Camilli, A.; Kawagishi, I. Mlp24 (McpX) of *Vibrio cholerae* implicated in pathogenicity functions as a chemoreceptor for multiple amino acids. *Infect Immun* **2012**, *80*,

3170-3178, doi:10.1128/IAI.00039-12.

9. Takahashi, Y.; Nishiyama, S.; Sumita, K.; Kawagishi, I.; Imada, K. Calcium Ions Modulate Amino Acid Sensing of the Chemoreceptor Mlp24 of *Vibrio cholerae*. *J Bacteriol* **2019**, *201*, e00779-00718, doi:10.1128/jb.00779-18.
10. Jeffery, C.J.; Koshland, D.E., Jr. *Vibrio cholerae hlyB* is a member of the chemotaxis receptor gene family. *Protein Sci* **1993**, *2*, 1532-1535, doi:10.1002/pro.5560020918.
11. Nishiyama, S.; Takahashi, Y.; Yamamoto, K.; Suzuki, D.; Itoh, Y.; Sumita, K.; Uchida, Y.; Homma, M.; Imada, K.; Kawagishi, I. Identification of a *Vibrio cholerae* chemoreceptor that senses taurine and amino acids as attractants. *Sci Rep* **2016**, *6*, 20866, doi:10.1038/srep20866.
12. Greer-Phillips, S.E.; Sukomon, N.; Chua, T.K.; Johnson, M.S.; Crane, B.R.; Watts, K.J. The Aer2 receptor from *Vibrio cholerae* is a dual PAS-heme oxygen sensor. *Mol Microbiol* **2018**, *109*, 209-224, doi:10.1111/mmi.13978.
13. Alexander, R.P.; Zhulin, I.B. Evolutionary genomics reveals conserved structural determinants of signaling and adaptation in microbial chemoreceptors. *Proc Natl Acad Sci U S A* **2007**, *104*, 2885-2890, doi:10.1073/pnas.0609359104.
14. Shiomi, D.; Zhulin, I.B.; Homma, M.; Kawagishi, I. Dual recognition of the bacterial chemoreceptor by chemotaxis-specific domains of the CheR methyltransferase. *J Biol Chem* **2002**, *277*, 42325-42333, doi:10.1074/jbc.M202001200.
